# ENGAGING OLDER ADULT LEARNERS AS HEALTH RESEARCHERS: PROGRAM OVERVIEW

**DOI:** 10.1093/geroni/igz038.1501

**Published:** 2019-11-08

**Authors:** Silvia Sörensen, Sandhya R Seshadri, Joyce Duckles

**Affiliations:** 1 University of Rochester Warner School for Education and Human Development, Rochester NY, United States; 2 University of Rochester School of Nursing, rochester, New York, United States; 3 Warner School for Education and Human Development, Rochester, New York, United States

## Abstract

Studies of aging rarely include the older adults themselves in the process of conceptualizing questions, implementing the research, and evaluating the results. To provide opportunities for community members to become engaged in research, researchers and community stakeholders developed “Engaging Older Adult Learners as Health Researchers (ENGOAL).” This program educates older adults from underserved and underresourced communities about geriatric health and research methods, enabling them to become Research Partners. Two cohorts of African-American seniors (N=21) aged 53-79 have participated or are currently participating in six months of weekly classes followed by 4-6 months of research apprenticeships. Content and structure of classes (covering qualitative and quantitative research approaches and language, salient health themes, and developing a research proposal) will be described. Challenges and successes in providing research apprenticeships (interviewing for a study on vision, reviewing recruitment materials, evaluating a mentorship program, and testing a diabetes management program) will be discussed.

